# Supplementary material for: Splice-Junction-Based Mapping of Alternative Isoforms in the Human Proteome
Source: Cell Rep. Author manuscript; Available in PMC 2020 Jan 15. (PMC6961840; doi:10.1016/j.celrep.2019.11.026)
Supplement: 2 [file NIHMS1546469-supplement-2.zip › DF1/PXD010154/Ovary/Ovary_19_COL4A5_GNPGPQGPPGRPGFQGLPGPEGPPGLPGNGGIKGEKGNPGQP.pdf]

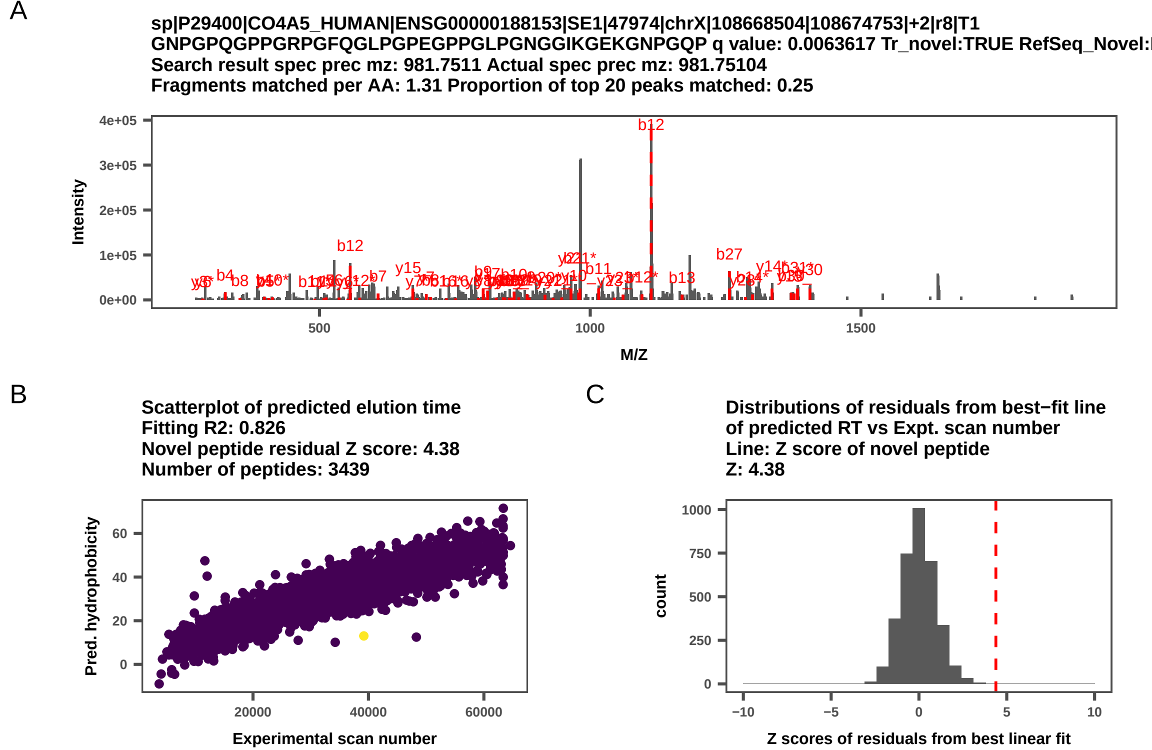
A
sp|P29400|CO4A5_HUMAN|ENSG00000188153|SE1|47974|chrX|108668504|108674753|+2|r8|T1
GNPGPQGPPGRPGFQGLPGPEGPPGLPGNGGIKGEKGNPGQP q value: 0.0063617 Tr_novel:TRUE RefSeq_Novel:
Search result spec prec mz: 981.7511 Actual spec prec mz: 981.75104
Fragments matched per AA: 1.31 Proportion of top 20 peaks matched: 0.25
Intensity
4e+05
3e+05
2e+05
1e+05
0e+00
M/Z
500
1000
1500
b12
b12
b27
b13
b11
b4
b8
b7
y15
B
Scatterplot of predicted elution time
Fitting R2: 0.826
Novel peptide residual Z score: 4.38
Number of peptides: 3439
Pred. hydrophobicity
60
40
20
0
Experimental scan number
20000
40000
60000
C
Distributions of residuals from best−fit line
of predicted RT vs Expt. scan number
Line: Z score of novel peptide
Z: 4.38
count
1000
750
500
250
0
Z scores of residuals from best linear fit
−10
−5
0
5
10
